# On impulsive integrated pest management models with stochastic effects

**DOI:** 10.3389/fnins.2015.00119

**Published:** 2015-04-21

**Authors:** Olcay Akman, Timothy D. Comar, Daniel Hrozencik

**Affiliations:** ^1^Department of Mathematics, Illinois State UniversityNormal, IL, USA; ^2^Department of Mathematics, Benedictine UniversityLisle, IL, USA; ^3^Department of Mathematics and Computer Sciences, Chicago State UniversityChicago, IL, USA

**Keywords:** birth pulse, stochastic component, probabilistic mixture, integrated pest management, impulsive differential equations

## Abstract

We extend existing impulsive differential equation models for integrated pest management (IPM) by including stage structure for both predator and prey as well as by adding stochastic elements in the birth rate of the prey. Based on our model, we propose an approach that incorporates various competing stochastic components. This approach enables us to select a model with optimally determined weights for maximum accuracy and precision in parameter estimation. This is significant in the case of IPM because the proposed model accommodates varying unknown environmental and climatic conditions, which affect the resources needed for pest eradication.

## 1. Introduction

One of the major challenges of the 21st century is providing food for an ever-growing population. Meeting this challenge requires a better job of protecting food crops from insects, as herbivorous insects are responsible for destroying one fifth of the world's total crop production annually (Sallam, 2013).

For virtually all pest species, eradication is biologically impossible or economically infeasible (Zhang et al., 2007). Hence, a variety of biological and chemical mechanisms need to be employed to control insect infestation. Individually, these techniques can control insect pest populations (Roland and Embree, 1995) but also can produce harmful environmental effects, e.g., damage to bird eggs through overuse of DDT, uncontrollable predator species proliferation. In many cases, long-term effects of biological or chemical intervention are virtually unknown (Reilly and Elderd, 2014). Used together, however, these strategies can control a pest population without the deleterious environmental effects (Van Lenteren and Woests, 1988; Song and Xiang, 2006). To meaningfully assess the outcomes of management techniques requires increasingly more accurate mathematical models that employ variations in environmental conditions and their impact on the interactions between plants and insects. In particular, exposure to extreme temperatures (potentially more frequent due to global warming) can affect, for example, lifespan, reproductive capacity, mobility and the overwintering strategies of insects (Hance et al., 2007; Robinet and Roques, 2010). Other effects include the geographic spread of agriculture-infesting pests as well as alien plant species of tropical origin (Kiritani, 2006). Likewise, exposure to extreme levels of precipitation (Masters et al., 1998; Rouault et al., 2006) or CO_2_ (Whittaker, 1999) over various time horizons have been shown to have a significant impact on populations of insect herbivores (Masters et al., 1998). From new technologies, such as drones, farmers are obtaining detailed micro-environmental information about their crops regarding insects, watering issues, and yields (Sharma, 2013; Doering, 2014). As pest control is becoming more data-driven, models must also evolve to reflect the precise yet increasingly complex data in order to produce effective and efficient pest control strategies.

In this paper, we address implementing biological (e.g., use of predator and disease) and chemical (e.g, use of pesticides) controls using impulsive differential equation (IDE) models by generalizing previous models to accommodate random fluctuations, not just periodic fluctuations, in the birth rate due to environmental and climatic factors such as temperature, light levels, and day length which may induce varying levels of egg production (Paulson et al., 2009). This approach enables us to extend our focus to incorporate random behavior, such as a random birth pulse, as well as *a priori* behavior that is unknown to the modeler.

The paper is organized as follows. In Section 2, we provide a survey of several IDE models implementing stage structure in the predator species and periodically varying environmental conditions with fixed birth rates for the prey species. In Section 3, we describe a more realistic model (Akman et al., 2013), which considers a random birth rate following an *a priori* distribution with a mean that replaces the fixed birth rates in prior models. Also in Section 3, we describe a model with a mixed birth rate pulse for the prey species (Akman et al., 2014). Finally in Section 4, we discuss future directions for modeling and model selection in the biological sciences.

## 2. Impulsive models

IDE provide a natural way to model integrated pest management systems. The periodic introduction of predators or diseased prey, application of pesticide, and births can be modeled as impulsive events. An important question for these models is determining the conditions either for which there are either **pest eradication solutions** to which all solutions converge (globally asymptotically stable solutions) or for which there are solutions where pest populations are kept at a sufficiently low non-zero level to prevent significant economic loss (**permanent solutions**). For mathematical details on the theory of IDE, we refer to the reader to the monograph (Lakshmikantham et al., 1989). For theoretical background on periodic solutions of such IDE, see (Bainov and Simenov, 1993).

KEY CONCEPT 1 **Pest Eradication Solution**

A solution for which the pest population eventually dies out.

KEY CONCEPT 2 **Permanent Solution**

A solution for which all populations in the system remain bounded by above and below by positive numbers.

One of the first impulsive models for managing a pest population using predatory biological control, due to Zhang et al. (2003, 2005), is given by the system

$$\begin{array}{l} {x^{\prime }}_{1}(t)=x_{1}(t)(b_{1}-x_{1}(t)-\alpha x_{2}(t)-r_{1}y(t))\\ {x^{\prime }}_{2}(t)=x_{1}(t)(b_{2}-x_{1}(t)-\beta x_{2}(t)-r_{2}y(t))\\ y^{\prime }(t)=y(t)(-b_{3}-dr_{1}x_{1}(t)+dr_{2}x_{2}(t)) \end{array}\}\;t\neq nT,$$

(1) $$\begin{array}{l} x_{1}(t^{+})=x_{1}(t)\\ x_{2}(t^{+})=x_{2}(t)\\ y(t^{+})=y(t)+p \end{array}\}\;t=nT,$$

where *b*_1_, *b*_2_ are growth rate parameters, α and β are competition factors, *r*_1_ and *r*_2_ are predation rates, *b*_3_ is a death rate parameter, *d* is a conversion rate from kills into new births, and *p* is the amount of the predator that is introduced into the population at periodic intervals of length *T*. Zhang et al. (2003, 2005) determined conditions for which the solutions of the model Equation (1) are globally asymptotically stable and for which the solutions are permanent.

Another form of biological control is the introduction of a disease into the pest population. Impulsive models of this type have been investigated by Zhang et al. (2007), Wang and Song (2008), and Sun and Chen (2009). The models in these papers separate the single pest population into a susceptible class and an infective class and periodically introduce new infective pests into the population. These models assume that while the susceptible population grows logistically, the infective population cannot cause excessive crop damage, and that the susceptible population can indeed destroy the crop. Moreover, there is a critical level for the susceptible population to cause crop damage. All three of these models assume non-linear rates of disease transmission (incidence rates) for the spread of the disease. The goal of these studies is to keep the susceptible population below the critical level by the introduction of infected pests into the population.

**Integrated pest management (IPM)** models incorporate two or more forms of control. Typically, IPM models only considering biological means of control may incorporate both a natural predator as well as a disease. Such models have been considered by Shi et al. (2009), Georgescu and Zhang (2010), Shi et al. (Shi and Chen, 2010), and Shi et al. (2013). In Shi et al. (2009), the incidence rate is linear in each variable (bilinear) and the predation follows a Holling type II functional response, which is a rational function that increases and eventually levels off as the prey population density increases. In this model, the infected pests and the predators are introduced into the system during separate impulsive events. The model due to Shi and Chen (2010) differs from this first one by assuming that infected pests and predators are introduced into the system simultaneously. The model in Georgescu and Zhang (2010) assumes a general non-linear incidence rate and a prey-dependent predation rate, both of which are increasing at a rate which is not increasing. In this model, the predator population is stage structured with an immature class and a mature class. Only the mature class acts as the predator and only attacks the susceptible pest. New predators and infected pests are periodically added to the system impulsively at the same time. All of these articles provide conditions for the global asymptotic stability of the susceptible pest eradication solution and for the the permanence of the system.

KEY CONCEPT 3 **Integrated Pest Management (IPM)**

The use of a combination of biological, chemical, and culture methods to control a pest population.

Shi et al. (2013) consider a model in which the prey species is separated into immature, mature susceptible, and mature infective classes. The disease incidence rate is bilinear, and the predation rate follows a Holling type II functional response. They consider two cases for the timing of the impulsive events for the introduction of infected pests and predators into the system: either the infected pests or the predators are introduced more frequently. Under both of these two timing conditions, Shi et al. (2013) determine conditions for global attractivity of the susceptible pest eradication solution and for the permanence of the system.

Chemical control (the application of pesticide) has also been used in IPM models along with biological controls. Models involving pesticide and natural predators have been studied by Hui and Zhu (2006), Song and Xiang (2006), Liu et al. (2006), Zhang et al. (2008), Baek et al. (2009), Wu and Huang (2009), and Gao and Tang (2011) among others. Hui and Zhu (2006) consider a prey-dependent consumption model in which pesticide is applied and the predator population is stage structured. In Song and Xiang (2006), the authors' model is a one-predator, two-prey, prey-dependent consumption model in which the predator is stage structured into an immature class and a mature class. In both models, the impulsive events of spraying pesticide, which is assumed only to affect the pest species, and the introduction of new predators into the system occur at the same time. In both papers, conditions are found for which the pest eradication solution is globally asymptotically stable and for which the system is permanent. Additionally, both Hui and Zhu (2006) and Song and Xiang (2006) consider variations of their models in which the intrinsic growth rates of the prey species are periodically varying.

Zhang et al. (2008) consider a model in which predation is modeled with a Beddington-DeAngelis functional response, which is dependent on both pest and predator populations. Pesticide, harmful to both species, and the introduction of new predators into the system both occur impulsively and periodically but at different times. The authors provide conditions for the global stability of the pest eradication solution and for the permanence of the system.

Wu and Huang (2009) analyze a model with multiple predators in which the predation follows an Ivlev-type functional response, which is commonly used to model predation by arthropods. The impulsive events of applying pesticide and the introduction of new predators occur periodically but at different times. Here, the authors give conditions for the stability and for the permanence of the system. Gao and Tang (2011) study the dynamics of a predator-prey model based on the Lotka-Volterra model in the application of pesticide and the introduction of new predators occur periodically during the same impulsive event.

Models involving pesticide and disease in the pest have been studied by Jiao and Chen (2006), Georgescu and Morosanu (2007), and Pang and Chen (2008). Jiao and Chen (2006) consider a model for IPM with an susceptible class and an infective class for the pest in which the incidence rate of disease is non-linear, and the two impulsive effects of spraying pesticide and introducing new infected pests occur periodically at the same time. Pang and Chen (2008) consider a similar model in which the disease incidence rate is bilinear and in which microbial pesticide is applied rather than chemical pesticide. In this model the impulsive event of introducing new infected pests is followed by the impulsive application of the microbial pesticide during each period. Georgescu and Morosanu (2007) develop a more general model in which the expression for the growth rate of the susceptible pests in the absence of infection generalizes logistic growth and the expression for the force of infection generalizes typically used linear and non-linear functions. The impulsive effects of spraying pesticide and introducing new infected pests occur periodically but at different times. Each of these papers provides conditions for the global asymptotic stability of the susceptible pest eradication solution and for the permanence of the system.

Yongzhen et al. (2010) study an impulsive model which incorporates natural predators, disease, and pesticide. The disease incidence is bilinear, and a Holling type II functional response is used for the predation. The impulsive effects of spraying pesticide and adding both new predators and infected pests to the system occur in a single impulsive event. The authors give conditions for the global asymptotic stability of the susceptible pest eradication solution and for permanence of the system.

The models we have discussed so far depend on impulsive events that occur periodically. Another class of impulsive models for IPM are state-dependent models in which impulsive control events occur when the pest population achieves an economic threshold level. These models have been extensively studied by Tang et al. (2005), Tang and Cheke (2005, 2008), Liang and Tang (2010), Tang et al. (2010), Gao and Tang (2011), and Zhao et al. (2014). For the sake of this review, we will focus our discussion on impulsive IPM in which the impulsive events occur periodically rather than according to state-dependent conditions.

Another impulsive event that may occur in IPM systems is the birth of the pest species via a birth pulse. For some animal species, individuals are born once per year or season rather than continuously. Two studies incorporating a birth pulse in an impulsive model are due to Roberts and Kao (1998) and Tang and Chen (2002). Roberts and Kao (1998) consider a the dynamics of a model for a fatal infectious disease for a wild animal population with a birth pulse. Tang and Chen (2002) study the dynamics of a single-species stage structured model with a birth pulse. Both Beverton-Holt (rational) and Ricker (exponential decay) type density dependent birth pulse functions are used in their model. The authors' models in Akman et al. (2013) and Akman et al. (2014) utilize birth pulses for the prey species.

## 3. Impulsive models with stochastic effects and mixed birth pulses

In Song and Xiang (2006), Song and Xiang consider a one-predator, two-prey impulsive IPM model in which the intrinsic growth rate of the prey varies periodically to incorporate seasonal variations for which the environmental and climatic conditions follow a predictable pattern. Similarly, Hui and Zhu (2006) consider a similar model with one prey species with a periodically varying intrinsic growth rate. In Akman et al. (2014) and Akman et al. (2013), Akman et al. include the behavior of the models in Hui and Zhu (2006) and Song and Xiang (2006) as particular instances. Here, the authors propose extending the models given in Hui and Zhu (2006) and Song and Xiang (2006) by constructing a parameterized set of models whose realization has the flexibility to accommodate the randomness in the intrinsic growth rate of the pest species. This randomness may be due to varying environmental and climatic conditions. The **stochastic** parameter selection mimics the implicit variability of these natural conditions. Thanks to the flexibility introduced with this approach, the resulting model allows for more realistic and accurate prediction of the optimal pesticide level, as explained below, needed for total eradication or economically feasible control of the pests. Just as Song and Xiang (2006) incorporates seasonal variation through the intrinsic growth rate parameter of the prey, the authors in Akman et al. (2014) and Akman et al. (2013) incorporate random variation through parameters in the birth pulses for the prey. Specifically, values for these birth rate parameters are chosen randomly from various probability distributions.

KEY CONCEPT 4 **Stochasticity**

Stochasticity allows greater flexibility in modeling by incorporating random variation over selected parameters.

By further incorporating stochastic parameters in the birth-pulse function, the proposed mixture model accommodates not only a broad range of birth rates but also different population growth behavior of the pest species. We achieve this by introducing a probabilistic mixture of different birth-pulse functions with stochastic weights that optimize the overall efficacy of the model.

In statistical modeling theory, it is well-known that the mixture modeling approach produces flexible models with good statistical and probabilistic properties. This is especially useful when *X* and *Y* are two competing random variables where unobserved heterogeneity that exists in the data collection process results in lack of fitness. Specifically, if 0 < π < 1 and *f_X_*(*x*) and *f_Y_*(*x*) are the probability density functions (pdf) of the random variables (r.v.) *X* and *Y*, respectively, then the pdf of the r.v. *Z* produced by the mixture between *X* and *Y* is given by

(2) $$f_{Z}(x)\;=\;(1-\pi )f_{X}(x)+\pi f_{Y}(x),x>0.$$

Our **model** that includes a set of external effects such as environmental and climatic conditions of a more random nature takes its inspiration from the approach given by Equation (2). We use two Beverton-Holt type birth-pulse functions, $B_{1}(t)=\frac{b_{1}x_{2}(t)}{q_{1}+x_{1}(t)+x_{2}(t)}$ and $B_{2}(t)=\frac{b_{1}x_{2}(t)}{q_{2}+x_{1}(t)+x_{2}(t)}$, which are widely used population density dependent birth-pulse functions Tang and Chen (2002). Additionally, we introduce further stochasticity by considering *b*_1_ and *b*_2_ as random variables. The impulsive effect for the immature prey in the model used in the stochastic version of System (1) in Akman et al. (2014) is defined by

(3) $$X_{1}(t^{+})=(x_{1}(t)+\pi B_{1}(t)+(1-\pi )B_{2}(t))(1-E),$$

for *t* = *nT*, where 0 < π < 1 is the mixing parameter, *T* is the frequency of the periodically occurring impulsive events, and *n* is any positive integer.

KEY CONCEPT 5 **Class of Models**

Various related models can be constructed by taking weight averages of different model components.

The concept of mixing birth-pulse functions with a mixing weight, π that produces an optimal birth pulse instantaneously followed by pesticide application is a novel concept. Previously discussed studies have reached conclusions based upon commitments to a singular birth-pulse function. The quality of their results is limited by its contingency upon the assumption that the chosen pulse function is the correct one. In a way, having confidence in a particular pulse function and then building a model based on it produces accurate results only within the realm of the cases where the assumed singular pulse function is applicable and appropriate. However, it seems reasonable to deduce that multiple pulse functions may be simultaneously at work, even during short periods of time due to ever-changing temporal and spatial environmental conditions and the resulting reproductive behavior. Hence the limited applicability of models with a single birth-pulse function may easily be rendered inefficient, if not unrealistic. With our approach, the birth-pulse parameter is employed in such a way that it reflects varying birth behavior, which may be due to climatic, environmental, ecological, or behavioral inconsistencies, among others. It is vital to emphasize that we are not attempting to directly model these inconsistencies but rather their effect on birth-pulse behavior. The optimal value of the new parameter π is determined based on the value of the parameter *E*, which measures the efficacy of the pesticide. The birth-pulse function weight π is obtained by minimizing the variation of *E*. Specifically, we examine three different levels of average birth rate, identifying low, medium, and high birth rate of the pest species. Then for each level, we investigate the model's efficiency under different stochastic *a priori* settings. For instance, an exponential prior distribution is employed to mimic the case where small birth pulses are more prevalent while large pulses are rare but possible. Even the non-informative case, where any birth-pulse is as likely as any other, is included in our study by employing a uniform distribution.

Here the ultimate goal is to determine the model with the smallest variation in *E*, which yields the model with most consistent eradication success rate. This, in turn, translates to designing a strategy in pest management by producing the most reliable model and associated parameter set that has the greatest chance of eradication or optimally controlled pest level. The possible noise, as in any stochastic approach, is handled by optimizing the eradication parameter under varying values of the birth-pulse, all centered around a plausible average value intended to accommodate potential noise. This assures that the constructed mixture model provides the most consistent values for pesticide use. We further consider the nature of the birth-pulse, which varies due to possible external conditions, in addition to the varying birth-pulse rate. Normally, the uncertainty of the external factors may prevent the correct determination of the conditions under which complete eradication or permanence occurs. Assuming the nature of pulse may follow *B*_1_(*t*), *B*_2_(*t*), or any weighted combination of these two birth-pulses, the model given by System (1) in Akman et al. (2014) has the added flexibility for accurately determining pest eradication or permanence. In fact, the approach we are introducing here by mixing pulse functions may be easily generalized by defining

(4) $$X_{1}(t^{+})=(x_{1}(t)+\sum_{k=1}^{k=n}\pi_{k}B_{k}(t))(1-E),$$

where $\sum_{k=1}^{k=n}\pi_{k}=1$. Clearly, replacing the mixed birth-pulse component of System (1) in Akman et al. (2014) with Equation (4) will provide an accurate fit under a wide spectrum of external conditions due to its generalized structure.

## 4. Concluding remarks and future directions

Model selection is well-established as a basic tool in select biological disciplines. As modeling becomes a more widespread practice in the life sciences and biomathematical sciences, researchers need reliable tools to calibrate models against ever more complex and detailed data, and hence model selection is now frequently employed as a way to identify the model that is best supported by the data (referred to as the *best model*) from among the candidate set. In other words, it can be used to identify the hypothesis that is best supported by observations. The process itself involves an accurate and precise estimation of modeling parameters. In fact parameter estimation is a key issue in computational and systems biology, as it represents the crucial step to obtaining quantitative and even qualitative information from dynamical models of biological systems.

Because of its very nature, the complexity in modeling arises due to its two main components: selecting the right model and then estimating the model parameters accurately and precisely. None of the commonly used modeling tools target both of these two components. For instance optimization tools such as simulated annealing, Monte Carlo Markov Chain (MCMC), or Akaike Information Complexity (AIC) require distributional assumptions some of which are not realistic in biomathematical modeling. Implementing genetic algorithms for model selection as well as parameter estimation is a focus of our future work. When coupled with sensitivity analysis, genetic algorithms are expected to produce models superior to existing models approached both in accuracy and precision.

The general approach proposed here has a unique advantage of being applicable in a wide spectrum of fields such as modeling in ecology or epidemiology among many others where the underlying model eventually depends on the choice of a function selected from a set of candidate functions. Furthermore, in particular, this approach produces more realistic results as to the resources necessary to ensure a high probability of pest eradication. This is important in cases in which the prey species is especially sensitive to changes in the potency of the pesticide. In fact, our modeling technique is versatile enough to be implemented even in cases with limited or no *a priori* information for birth rate distributions.

As models become more complex due to the incorporation of stochastic effects and weighted components that contain *a priori* information, the processes of model selection and subsequent parameter estimation become more computationally difficult. Therefore, using powerful numerical techniques become vital in the modeling process. However, most commonly used numerical methods are neither suitable nor computationally efficient for these increasingly complex models. Moreover, commonly used traditional models lack the ability to assess the appropriateness of a particular choice of model. Consequently, a poor modeling choice leads to meaningless parameter estimation. An ultimate validity of a model lies in how well it predicts outcomes of interest in real-life settings. However, before a practical verification of a model can be considered, it has to be constructed, tested, and verified in simulated numerical environments. Only then should verification of a model using real-life data be attempted. We see the practical usability of our approach as the next natural step in the modeling process. In fact once the data are acquired, the method by which the model is justified is a research project by itself due to the complex nature of the parameter estimation process. Intelligent optimization methods become especially noteworthy when verification of these methods, or any other modeling approach, use real-life data. Evolutionary computing methods, such as genetic algorithms, have the added advantage of being able to assess the validity of the optimal model along with the ability of producing accurate and precise estimates under complex modeling assumptions. Hence, using evolutionary computing methods for models with stochastic effects is one of the next meaningful steps to explore.

### Conflict of interest statement

The authors declare that the research was conducted in the absence of any commercial or financial relationships that could be construed as a potential conflict of interest.
